# Effects of Different Pollination Methods on Oilseed Rape (*Brassica napus*) Plant Growth Traits and Rapeseed Yields

**DOI:** 10.3390/plants11131677

**Published:** 2022-06-24

**Authors:** Jianwen Zhang, Songchao Zhang, Jiqiang Li, Chen Cai, Wei Gu, Xiaohui Cheng, Haohan Wang, Xinyu Xue

**Affiliations:** 1 Nanjing Institute of Agricultural Mechanization, Ministry of Agriculture and Rural Affairs, Nanjing 210014, China; zjw1967@163.com (J.Z.); caichen@caas.cn (C.C.); guwei01@caas.cn (W.G.); 2 Zhangye Academy of Agricultural Sciences, Zhangye 734000, China; zynksljq@163.com (J.L.); zywhh8@sina.com (H.W.); 3 Sino-USA Pesticide Application Technology Cooperative Laboratory, Nanjing 210014, China; 4 Oil Crops Research Institute, Chinese Academy of Agricultural Sciences, Wuhan 430062, China; chengxiaohui@caas.cn

**Keywords:** hybrid oilseed rape, seed production, pollination method, unmanned agricultural aerial system, growth trait, yield

## Abstract

Pollination success is essential for hybrid oilseed rape (OSR, *Brassica napus*) seed production, and the pollination method has some influences on the OSR plant growth traits. In order to explore the roles of different pollination methods, four pollination methods of “unmanned agricultural aerial system” (UAAS), “natural wind + UAAS” (NW+UAAS), “honeybee” (HB), and “no pollinators” (NP) were set in a hybrid OSR field to investigate their effects on OSR plant traits and rapeseed yields in this study. The control check (CK) area with natural wind (NW) pollination was set as a reference for comparison. The experiments were conducted continuously for 20 days during the OSR plant early to full-bloom stage. The results based on the evaluated OSR plants showed that the growth traits and the rapeseed yields exhibited some differences under different pollination methods. The average plant height under NP pollination was maximum, which was 231.52 cm, while the average plant heights under the other pollination methods exhibited nearly no difference. Except for the HB pollination, the average first-branch heights of the evaluated plants all exceeded 100 cm under the other pollination methods. The average once branch quantity of all the evaluated plants under different pollination methods was 5–7. The average number of effective siliques per plant varied greatly. The average quantity of effective siliques in each OSR plant was about 160 under UAAS, NW+UAAS, and NW pollination, about 100 under HB pollination, and only 2.12 under NP pollination. The thousand-rapeseed weight was 7.32 g under HB pollination, which was the highest of all of the pollination areas. In terms of rapeseed yield, the average rapeseed yields per plant were all more than 10 g, except for the one under NP pollination; the yield per hectare was highest under NW+UAAS pollination, reaching 4741.28 kg, and the yield under NP pollination was lowest, which was only 360.39 kg. The research results provide technical support for supplementary pollination in hybrid OSR seed production.

## 1. Introduction

Oilseed rape (OSR, *Brassica napus*) is the third-most-important oil crop in the world [1,2]; high-quality OSR oil meets the criteria of the most demanding nutritionists, and it is used as a feed pellet for livestock species and resources for certain industrial products [3,4,5,6]. Therefore, the OSR planting areas are expanding rapidly, especially those of hybrid OSR [7]. Seed production with the cross-pollination hybrid’s vigor is an important means for high OSR seed yields [8]. During OSR seed production, the pollen from the male OSR plants spreads onto the pistils of the female OSR plants, which bear siliques that then ripen into seeds. Successful OSR pollen spreading guarantees seed production [9,10]. The traditional pollination methods include natural wind (NW), manual, and insect pollination. NW pollination is the most economical method, but also the most uncertain, because the velocity and the direction of the natural ambient wind are not controlled [2]. Manual pollination is inefficient and is not suitable for large-scale field OSR seed production. Insect, especially honeybee (HB), pollination is vital for high yields of OSR [11,12,13,14,15], and HBs have been considered as the most important insect pollinator [16,17]. In recent years, as the level of agricultural production mechanization has improved, more and more new intelligent agricultural machinery has been used in agriculture, of which the unmanned agricultural aerial system (UAAS) is a typical representative. The UAAS has been used in agriculture for crop protection [18], remote sensing [19], particle fertilizing and seeding [20], etc. For OSR, the UAAS has also been used for *Sclerotinia sclerotiorum* control and protection, foliar fertilizer spraying, aerial rapeseed seeding, and supplementary pollination [2].

The average effective siliques per plant (AESPP), the average rapeseed yields per plant (ARYPP), and the thousand-rapeseed weight (TRW) are the most important factors concerning the rapeseed yield [21]. Meanwhile, the other OSR plant growth traits, such as the average plant height (APH), the average first branch height (AFBH), and the average once branch quantity (AOBQ), also affect the yield of hybrid OSR seeds [22]. Some scholars have conducted studies on OSR plant growth traits in field hybrid rapeseed production and the influences of these traits on the yield. Zhang et al. [23] analyzed the effect of different altitudes on agronomic traits, yield, and quality. Their results showed that, as the altitude increased, the APH, AFBH, and AOBQ decreased, the TRW increased, and the AESPP and the yield first decreased and then increased. Three types of *Brassica napus*, Dadi 95, Jinghua 165, and Shanyou 2, were chosen to study the influences of altitude on the agronomic and quality traits by Zhao et al. [24]. They found that altitude was positively correlated with the growth period, and significantly negatively correlated with the APH and ARYPP, with correlation coefficients of 0.519, −0.548, and −0.528, respectively. Similarly, according to Zhang et al. [23], the TRW increased as the altitude increased. Under the low-temperature and drought planting conditions in Gansu Province, China, Zhao et al. [25] set five treatments, flat planting with half-mulching by plastic film (FHP), ridge planting with half-mulching by plastic film (RHP), flat planting with complete-plastic-film mulching (FOP), ridge planting with complete-plastic-film mulching (ROP), and flat planting without mulching as a control check (CK), to explore the effects of the cultivation methods on the promotion of the growth traits and yield of rapeseed production. The results showed that the FHP, RHP, FOP, and ROP treatments all promoted the individual OSR plant growth, and the AESPP, the ARYPP, and the yield all increased. The ROP had the most advantages among the cultivation methods—the rapeseed yield was increased by 34.05% compared with the CK treatment, which was recommended as the high-efficiency cultivation mode for spring OSR seed production. In order to investigate the effect of preventing insect nets (PINs) on the OSR plant growth and hybrid rapeseed yield, Wen et al. [26] carried out experiments with PINs with different net densities, and the test results showed that the sunlight intensity decreased significantly and the rapeseed yield decreased as the PIN mesh density increased, while the PINs could improve the air moisturizing effect in the area covered by the PINs. Additionally, some other studies based on genetic analysis determined the factors that affect the OSR plant growth traits and rapeseed yield [22,27,28,29,30].

In summary, the above research conclusions indicate that the OSR plant growth traits and rapeseed yield would be affected by the attitude, cultivation, sunlight, temperature, etc. As mentioned above, successful pollination is essential for hybrid OSR seed production, which would provide sufficient high-quality seeds for large-scale planting. However, there is still a lack of research on the effect of pollination methods on the hybrid OSR plant growth traits and the rapeseed yield. Therefore, in this study, the authors aimed to investigate the effects of UAAS, NW+UAAS, HB, and NP pollination methods on the OSR plant growth traits and yield, screening out the best pollination method to provide a reference for hybrid OSR seed production.

## 2. Materials and Methods

### 2.1. Experimental Site, OSR Plant Characteristics, and Weather Conditions

The experimental site was located in the OSR field of Minle Agricultural Base (38.4692° N, 100.8674° E) in Zhangye City, Gansu Province, China. The trials were carried out from 1 July to 20 July 2021, which was the local season for OSR pollination. The OSR variety was Ganyouza 8 (Registration Number: GPD Rape (2017) 360100, Ministry of Agriculture and Rural Affairs of the People’s Republic of China) [31], which is a hybrid rapeseed combination of ZS11 (male OSR plant) and 283B (female OSR plant). The OSR plant was transplanted into the field manually in April 2021. The main characteristics of the OSR plant and the weather conditions are shown in Table 1.

### 2.2. Experimental Materials

**Figure 1:**
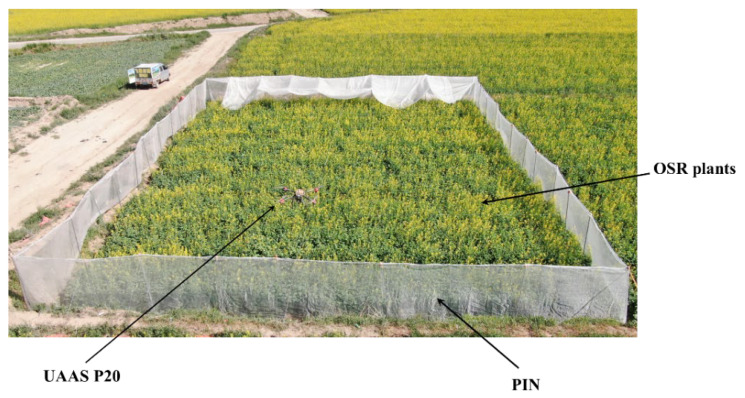
The UAAS P20 flying in the UAAS pollination area.

A 60-mesh PIN with a mesh size of 0.25 mm×0.25 mm, a hive of about one- thousand honeybees (HB), and the UAAS of P20 (Guang Zhou XAG Co., Ltd., Guangzhou, China, as shown in Figure 1) were used in the pollination tests. The PINs were used to surround isolated test areas, the HBs were used for insect pollination within the isolated area, and the UAAS was used to fly above the OSR plants in the UAAS pollination and NW+UAAS pollination areas. The OSR plants in all pollination areas were managed with the same water and fertilizer.

The UAAS P20 is a four-rotor UAAS with a real-time kinematic Global Positioning System (RTK-GPS). It flies fully autonomously with the routes planned by the mobile app, and the flight speed and flight height can be set in the mobile app. The main technical parameters are shown in Table 2.

### 2.3. Experiment Design

PINs are often used in field crop production, which could effectively prevent insects, and adjust the temperature, humidity, and other microclimates in the shed [32,33]; meanwhile, PINs can also significantly reduce natural wind ventilation [34,35]. In this study, we ignored the adjustment effect of PINs on the microclimate in the pollination areas, and chose a PIN with a smaller mesh size (0.25 mm × 0.25 mm) than the one used in [34,35], which was believed to further reduce the ventilation rate of natural wind in the field, to achieve the expected effect of isolating from natural wind. In addition, it should be noted that, although the hybrid OSR at the experimental site mainly relied on natural wind pollination, there would inevitably be a few wild HBs in the field naturally, which would have a negligible impact on the study results. Therefore, wild HBs were not considered in this study.

**Figure 2:**
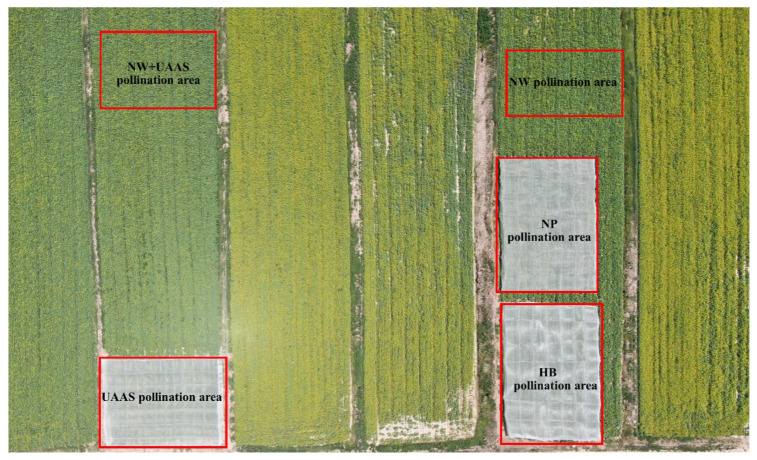
The whole experimental field and each pollination area setting (top view).

The whole experimental field was divided into five pollination areas: UAAS pollination area, NW+UAAS pollination area, HB pollination area, NP pollination area, and NW pollination area (CK area), shown in Figure 2. Before the OSR plant bloomed, the UAAS pollination areas, the HB pollination area, and the NP pollination area were surrounded by the selected PINs to form enclosed spaces with a size of 29 m × 20 m × 2.5 m. Thus, the wild HBs could be well-blocked from entering these pollination areas to minimize their influences on the experimental results. The NW+UAAS pollination area and the NW pollination area also had a size of 29 m × 20 m. In the UAAS pollination area, the spread of OSR pollen was mainly affected by the UAAS wind field, ignoring the weak natural wind compared with the UAAS wind during the short pollination test period every day. In the NW+UAAS pollination area, the pollen spread was affected by both the UAAS wind field and the natural wind; that is, the pollen spread was mainly affected by the UAAS wind field during the pollination test each day when the UAAS was flying above the OSR plants, and by natural wind as the pollination test finished. In the HB pollination area, the spread of OSR pollen was mainly affected by the HBs at all times. In the NW (CK) pollination area, the spread of OSR pollen was mainly affected by the natural wind at all times, which is the popular pollination method locally. In the NP pollination area, there was almost no medium for OSR pollen spread due to the isolated enclosed space.

In the pollination tests, the UAAS was planned automatically to fly perpendicular to the OSR plant rows repeatedly and alternately in the NW+UAAS and UAAS pollination areas, and fly above the OSR plants during the experimental period each day from 9:00 AM to 11:00 AM, which is the agronomic time for OSR plant pollination. Before the UAAS flew above the OSR plants in the UAAS pollination area, the top PIN was first uncovered manually, and then covered to restore the isolated enclosed pollination area after the UAAS supplemental pollination was finished. The UAAS flew at a speed of 4.0 m/s and height of 2.5 m, which were the optimized flight parameters according to Zhang et al. [2]. The HBs were added to the HB pollination area on 1 July.

The trials lasted for twenty days from 1 July to 20 July 2021.

### 2.4. OSR Plant Growth Trait Definitions and Rapeseed Yield

The average plant height (APH), the average first branch height (AFBH), the average once branch quantity (AOBQ), the average effective siliques per plant (AESPP), the thousand-rapeseed weight (TRW), and the average rapeseed yield per plant (ARYPP) were the indexes used to evaluate the OSR plant growth traits. They are defined below [36].

APH is the average height of the measured OSR plants—the OSR plant height: the length from the cotyledon node to the top of the main inflorescence of the plant, cm.

AFBH is the average height of the first branch on the measured plants—the first branch height: the height from the cotyledon node to the first effective branch at the bottom of the main stem, cm.

AOBQ is the average once branch quantity of the measured plants—the once branch quantity: the quantity of once branches with more than one effective silique on the main stem.

AESPP refers to the total number of siliques containing more than one normal seed in the whole plant.

TRW: use a sample divider or other methods to count 1000 rapeseeds for three samples and weigh them, and take the average weight of the three samples as TRW. In this study, three groups of 1000 relatively plump rapeseeds in each pollination area were selected manually for TRW.

ARYPP: thresh the seeds of the evaluated OSR plants, dry and weigh them, and take the average seed weight as ARYPP, g.

**Figure 3:**
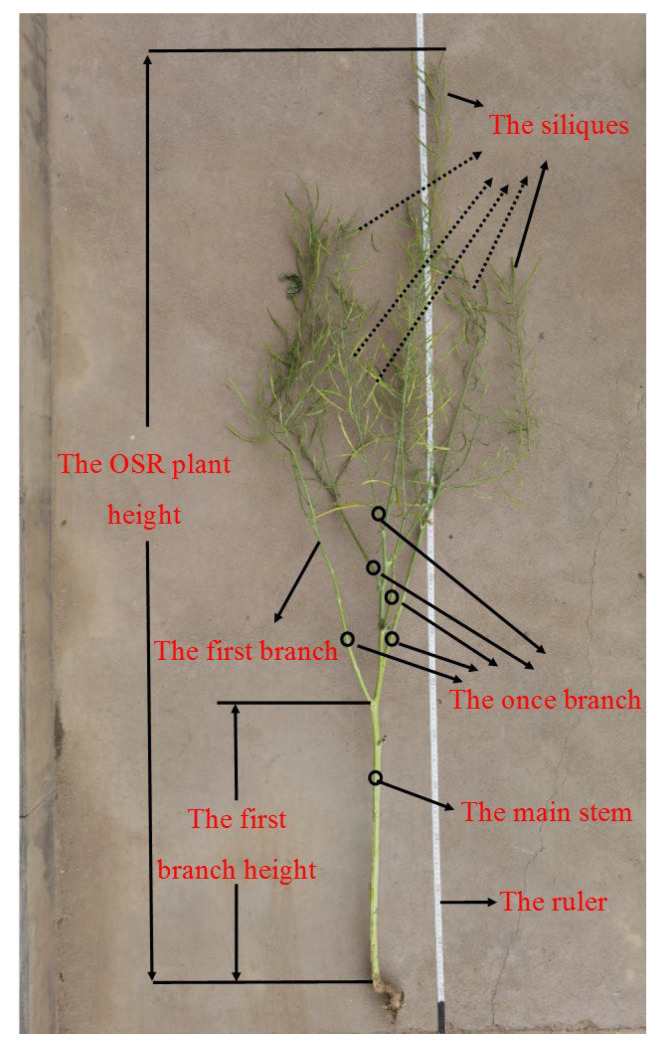
The OSR growth traits shown on a measured plant.

The OSR plant growth traits are shown in Figure 3 to provide a better understanding.

The rapeseed yield per hectare was obtained by a field yield test—the specific method was to select three replicates of 3 m × 3 m areas randomly from each pollination area to measure the rapeseed yield of each replication area, then take the average yield of the three replicates as the yield of a 3 m × 3 m area, and convert it to the rapeseed yield per hectare.

For the above traits, except for the TRW and rapeseed yield, three replicates of twenty OSR plants in each pollination area were sampled randomly and measured. First, the arithmetic mean value of each growth trait in every replicate was calculated; then, the average value of the arithmetic values of each growth trait in the three replicates was calculated to evaluate the growth traits and the rapeseed yield. The software OriginPro 9.0 (OriginLab, Northampton, MA, USA) was used to analyze the data.

## 3. Results

**Figure 4:**
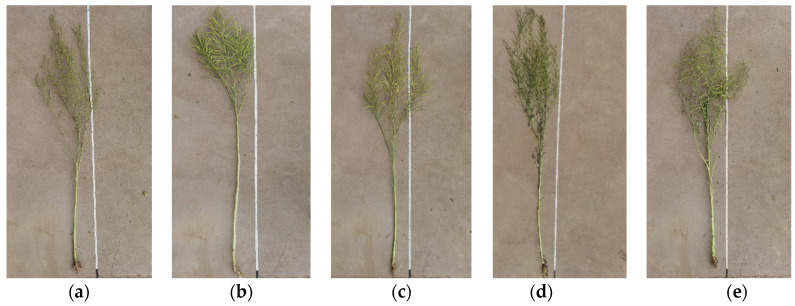
The measured OSR plants; (**a**) OSR plant from the UAAS pollination area; (**b**) OSR plant from the NW+UAAS pollination area; (**c**) OSR plant from the HB pollination area; (**d**) OSR plant from the NP pollination area; (**e**) OSR plant from the NW pollination area.

Figure 4 shows some evaluated OSR plants from different pollination areas that we measured. It could be seen that there were some trait differences between the shown plants. The APH, AFBH, AOBQ, and AESPP were the indexes used to quantitatively compare the OSR plant growth traits [37], and the rapeseed yield per hectare was statistically analyzed [38] under different pollination methods.

The OSR plant trait parameters and the rapeseed yield in the CK (NW pollination) area were used as references; the change range of each plant trait parameter and the yield in other pollination areas were compared with the reference. The calculation formula is as follows.

(1) $$\theta_{x}=\frac{a_{x}-b_{x}}{b_{x}}\times 100\%$$

where *x* represents the APH, AFBH, AOBQ, AESPP, TRW, ARYPP, or the yield per hectare, a  represents the OSR plant parameter or yield obtained from other pollination areas, b represents the OSR plant parameter or yield obtained from the NW pollination area, and θ is the change range, %.

### 3.1. OSR Plant Growth Traits and Comparisons

#### 3.1.1. APH Analysis

**Figure 5:**
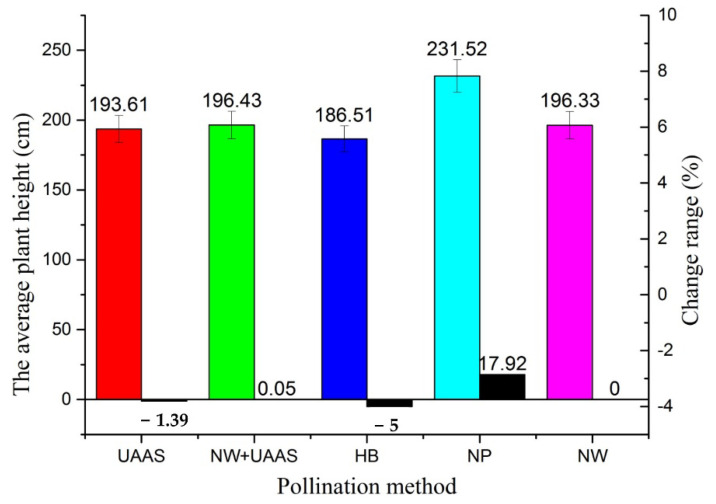
The APHs of different pollination areas and APH change ranges. Note: UAAS—unmanned agricultural aerial system; NW+UAAS—natural wind + UAAS; HB—honeybee; NP—no pollinators; NW—natural wind; APH—average plant height.

The APH of each pollination area and the APH change range are shown in Figure 5. From Figure 5, it can be seen that the APH with the NP pollination method was highest, which was 231.52 cm. The APHs with different pollination methods did not vary much; all were about 190 cm, except for that of NP pollination. Compared with the NW pollination method, the APHs of the UAAS and HB decreased, while the APHs of NW+UAAS and NP increased, and the change ranges were −1.39%, −5%, 0.05%, and 17.92%, respectively.

#### 3.1.2. AFBH Analysis

**Figure 6:**
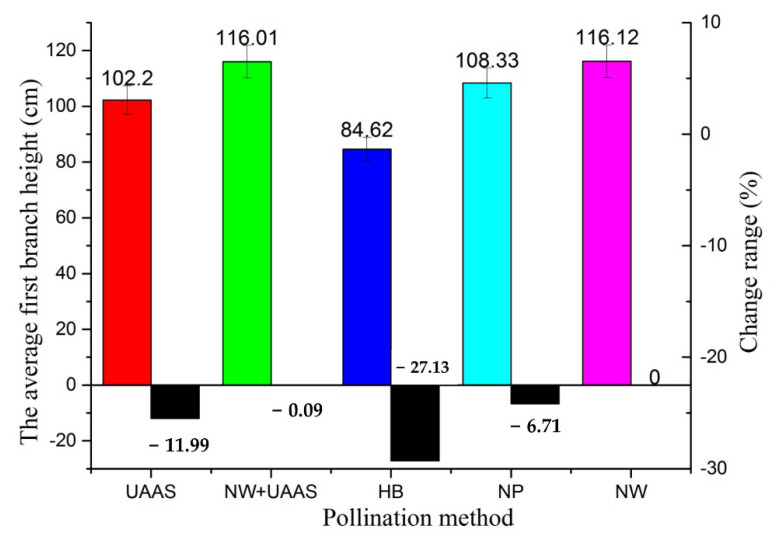
The AFBHs of different pollination areas and AFBH change ranges. Note: UAAS—unmanned agricultural aerial system; NW+UAAS—natural wind + UAAS; HB—honeybee; NP—no pollinators; NW—natural wind; AFBH—average first branch height.

Figure 6 shows the AFBH results of different pollination areas. It can be seen that the AFBHs almost all exceeded 100 cm, except for that of HB pollination. The AFBHs of NW+UAAS and NW were very close, which were 116.01 cm and 116.12 cm, respectively. Compared with the NW pollination method, the AFBHs of other pollination methods all decreased, and the change ranges were −11.99%, −0.09%, −27.13%, and −6.71%, respectively.

#### 3.1.3. AOBQ Analysis

**Figure 7:**
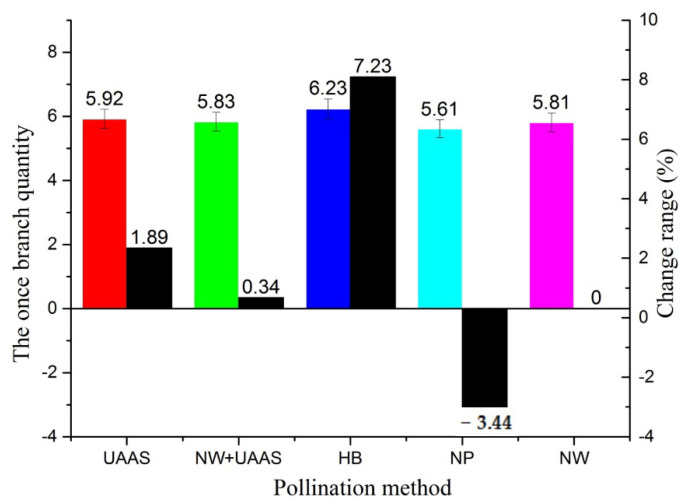
The AOBQs of different pollination areas and AOBQ change ranges. Note: UAAS—unmanned agricultural aerial system; NW+UAAS—natural wind + UAAS; HB—honeybee; NP—no pollinators; NW—natural wind; AOBQ—average once branch quantity.

The AOBQ of each pollination area and the AOBQ change range are shown in Figure 7. The AOBQs of the plants under different pollination methods were all 5–7. The AOBQ of the HB pollination method was highest, at 6.23, and that of NP was lowest. The change ranges did not vary greatly; only that of NP decreased by −3.44% compared with the NW pollination method.

#### 3.1.4. AESPP Analysis

**Figure 8:**
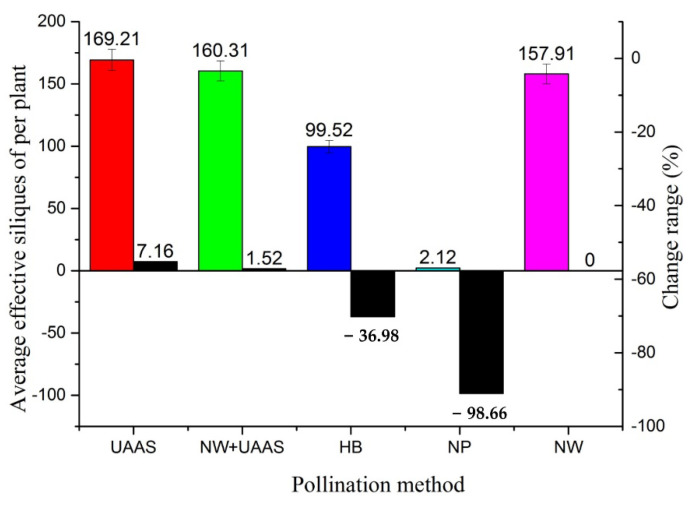
The AESPPs of different pollination areas and AESPP change ranges. Note: UAAS—unmanned agricultural aerial system; NW+UAAS—natural wind + UAAS; HB—honeybee; NP—no pollinators; NW—natural wind; AESPP—average effective siliques per plant.

The AESPPs of different pollination areas had significant differences, as shown in Figure 8. The AESPP of the NP pollination area was lowest, at only 2.12, while those of the other pollination methods were close to or over 100, which indicated that the pollination method had a great influence on the AESPP. The AESPPs of the UAAS, NW+UAAS, and NW pollination methods were very close, at 169.21, 160.31, and 157.91, respectively; the AESPP of HB was second-lowest, at 99.52, between the AESPPs of NW+UAAS and NW. Compared with the NW pollination method, the AESPPs of UAAS and NW+UAAS increased, while the AESPPs of HB and NP decreased; the change ranges were 7.16%, 1.52%, −36.98%, and −98.66%, respectively.

#### 3.1.5. TRW Analysis

**Figure 9:**
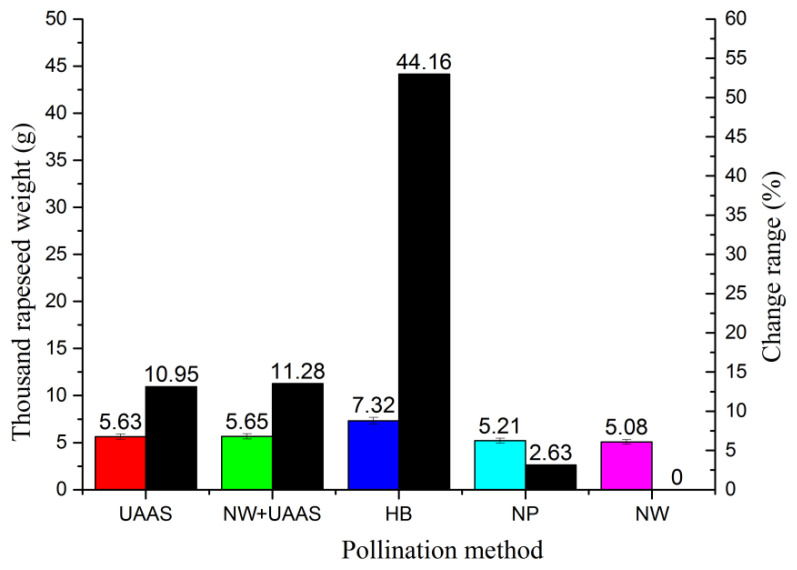
The TRWs of different pollination areas and TRW change range. Note: UAAS—unmanned agricultural aerial system; NW+UAAS—natural wind + UAAS; HB—honeybee; NP—no pollinators; NW—natural wind; TRW—thousand-rapeseed weight.

Most of the TRWs were between 5 g and 6 g; only the TRW of the HB pollination method exceeded 7 g, as shown in Figure 9. The TRW of NW pollination was lowest; the other TRWs all increased compared with the NW pollination method, with change ranges of 10.95%, 11.28%, 44.16%, and 2.63%, respectively.

### 3.2. Rapeseed Yield

#### 3.2.1. ARYPP Analysis

**Figure 10:**
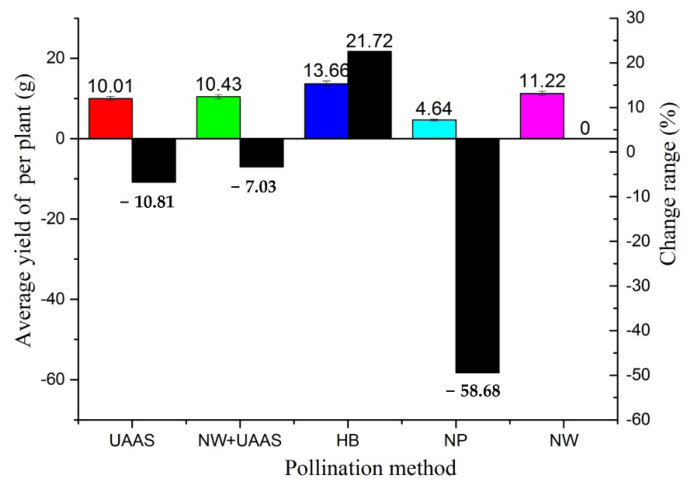
The ARYPPs of different pollination areas and ARYPP change ranges. Note: UAAS—unmanned agricultural aerial system; NW+UAAS—natural wind + UAAS; HB—honeybee; NP—no pollinators; NW—natural wind; ARYPP—average rapeseed yields per plant.

The ARYPP results are shown in Figure 10. The ARYPP of the HB pollination method was heaviest, at 13.66 g while, that of NP was lightest, at 4.64 g. The ARYPPs of UAAS, NW+UAAS, and NW with wind as a pollen-spreading medium were close. Compared with the NW pollination method, the ARYPPs of the UAAS, NW+UAAS, and NP all decreased; only the ARYPP of HB increased—the change ranges were −10.81%, −7.03%, −58.68%, and 21.72%, respectively.

#### 3.2.2. Rapeseed Yield per Hectare

**Figure 11:**
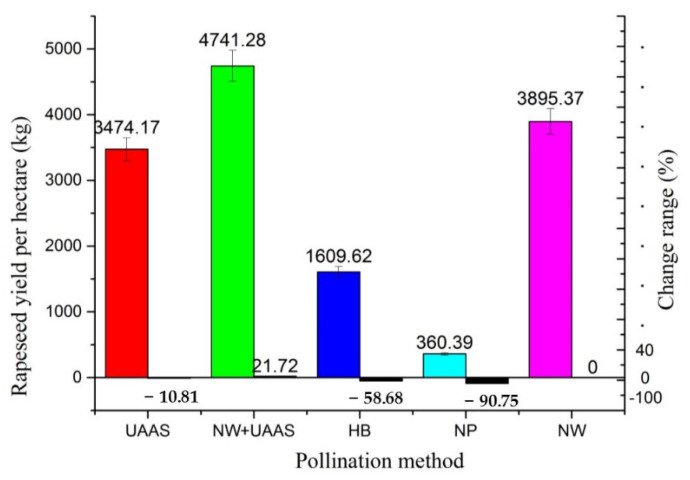
The rapeseed yield per hectare in each pollination area and the yield change ranges. Note: UAAS—unmanned agricultural aerial system; NW+UAAS—natural wind + UAAS; HB—honeybee; NP—no pollinators; NW—natural wind.

The rapeseed yield per hectare of each pollination area is shown in Figure 11. It can be seen that the rapeseed yield per hectare had significant variations between these areas. The maximum yield per hectare exceeded 4000 kg (NW+UAAS), while the minimum yield did not reach 400 kg (NP); the yield difference was more than ten times. This indicated that the rapeseed yield was highly and significantly affected by the pollination method. The rapeseed yield change ranges of the other pollination methods were −10.81%, 21.72%, −58.68, and −90.75%, respectively, compared with the NW pollination method.

## 4. Discussion

Hybrid OSR seed production is very important for large-scale OSR planting, and pollination is an essential technology for rapeseed production because the daily agronomic pollination time period is very short. In this paper, we investigated five different pollination methods to explore the influences of pollination on the OSR plant growth traits and the rapeseed yield in order to find out the optimal methods for improving the pollination efficiency. Through the assessments of the growth traits and the yields of the OSR plants (Ganyouza 8) after the experiments, generally, the five designed pollination methods had little influence on AOBQ, but had various degrees of influence on APH, AFBH, AESPP, TRW, ARYPP, and the rapeseed yield. Thus, it is believed that it is necessary to select and optimize a pollination method in order to ensure efficient rapeseed production, which is also the significance of this study.

### 4.1. Growth Traits

In the NP pollination area, the APH was highest, at 231.52 cm, while the AOBQ, the AESPP, and the ARYPP were lowest. The reason may be that the nutrients of the plant were mostly used for plant growth and less for silique formation due to the lack of a pollen-spreading medium.

In the HB pollination area, the AOBO, the TRW, and the ARYPP were highest, at 6.23, 7.32 g, and 13.66 g, respectively. Although the APHs did not vary greatly between different pollination areas, the APH was lowest, at 186.51 cm, in the HB pollination area. The results were the opposite of the OSR plant traits in the NP pollination area. This may be because the nutrients of the plant were mostly used for silique formation and less for plant growth due to the HBs’ continuous pollination.

In the UAAS, NW+UAAS, and NW pollination areas, the growth traits of APH, AFBH, AOBQ, AESPP, TRW, and the ARYPP were close. In these pollination areas, there was a same factor, “airflow”, whether natural wind or the wind field generated by the UAAS. Therefore, it was considered that the wind had a significant effect on the OSR plant growth traits.

The reason for the AESPP of the UAAS pollination area being highest may be that the OSR plants were disturbed by the UAAS wind field causing the pollen to float in the air [2], which would improve pollen spread to the female OSR plant pistils. The AESPP of the NP pollination area was only 2.12, which was the lowest of all of the pollination areas. This shows the importance of pollination in OSR silique growth—the AESPP reduced by 98.66% compared with the AESPP of NW pollination without a pollination medium.

### 4.2. TRW and Yield

TRW is an important index that reflects the fullness and quality of seeds. To determine the TRW, we counted 1000 rapeseeds for three samples from different pollination areas manually, and weighed them. The result showed that only the TRW of the HB pollination area exceeded 7 g; the TRWs of the other pollination areas were not greatly different. The TRW of the evaluated OSR plants from the HB pollination area was highest, similar to the ARYPP, which indicated that the HBs played a positive role in hybrid OSR seed production compared with the results in the NP pollination area.

From the perspective of rapeseed yield, the yield of the NP pollination area was very low, which was more than 90% lower than that of the CK area. This demonstrated the importance of efficient pollination for hybrid OSR seed production assurance once again. The very low rapeseed yield of the NP pollination area also proved the assumption in the experiment design that the selected PIN could reduce the ventilation rate of natural wind to a large extent, which ensured the reliability of the experimental results. By comparing the rapeseed yield of the UAAS and NW+UAAS pollination areas, the hybrid rapeseed yield increased under the combined effect of natural wind and the UAAS wind field, indicating that the environmental wind played a positive role in pollination, which would be also verified by the result of the rapeseed yield in the NW pollination area.

## 5. Future Work

Future research will focus on the hybrid OSR varieties and the economic analysis of different pollination methods. In this study, we choose Ganyouza 8 as the pollination object, and other hybrid OSR plant growth traits and rapeseed yields need to be studied. From the point of view of economic benefits, although the rapeseed yield under NW+UAAS pollination increased compared with the NW pollination method, the additional input of the UAAS and labor was required. Whether the cost of the input is higher than the value of the increased rapeseed yield or not is worth further research.

## Figures and Tables

**Table 1:** OSR plant characteristics and weather conditions.

Test Time	Growth Period	OSR Plant Mean Height (cm)	Row Proportion of Male to Female	Mean Wind Speed (m/s)	Mean Temperature (°C)	Mean Relative Humidity (%)
1 to 20 July 2021	Early to full stage of blooming	160 ± 10 (male) 150 ± 10 (female)	2:10	0.73 ± 0.20	24.75 ± 0.30	33.1 ± 1.85

**Table 2:** The main technical parameters of P20.

Items	Parameters
UAAS size	1262 mm × 1250 mm × 490 mm
Rotor diameter	36 cm
Battery capacity	18,000 mAh × 2
Flight speed	3–7 m/s
Flight height	0.5–3 m

## Data Availability

No new data were created or analyzed in this study. Data sharing is not applicable to this article.
